# Synthesis and Purification of Lipid-conjugated Fluorescent pH Sensors

**DOI:** 10.21769/BioProtoc.4694

**Published:** 2023-06-05

**Authors:** Wiebke Wiesner, Ronja Marie Kühnel, Thomas Günther Pomorski

**Affiliations:** 1 Department of Molecular Biochemistry, Faculty of Chemistry and Biochemistry, Ruhr University Bochum, Bochum, Germany; 2 Department of Plant and Environmental Sciences, University of Copenhagen, Frederiksberg, Denmark; # Contributed equally to this work

**Keywords:** Electrochemical gradients, Fluorescence, Labelled lipids, Liposome, Membrane transporter, pH sensor, Reconstitution

## Abstract

Lipid-conjugated pH sensors based on fluorophores coupled to lipids are a powerful tool for monitoring pH gradients in biological microcompartments and reconstituted membrane systems. This protocol describes the synthesis of pH sensors based on amine-reactive pHrodo esters and the amino phospholipid phosphatidylethanolamine. The major features of this sensor include efficient partitioning into membranes and strong fluorescence under acidic conditions. The protocol described here can be used as a template to couple other amine-reactive fluorophores to phosphatidylethanolamines.

Graphical overview

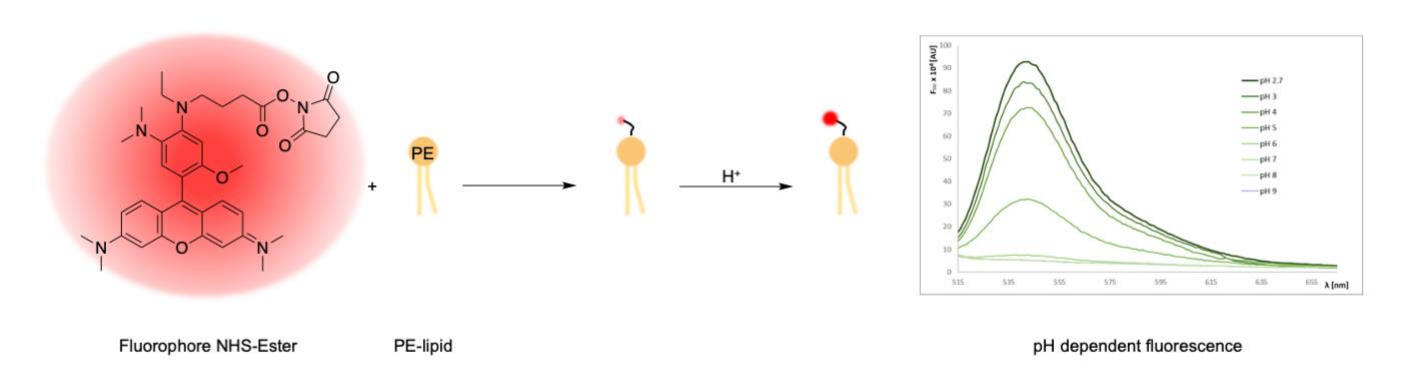

Synthesis of lipid-conjugated pH sensors based on amine-reactive fluorophore esters and the aminophospholipid phosphoethanolamine (PE)

## Background

Cells and organelles depend on electrochemical gradients across their membranes, which are maintained through ATP-driven, membrane-embedded transporter proteins. Transport of ions across cellular membranes can be primary or coupled to drive the uptake or export of other solutes. Perturbations of the highly regulated transport systems are the cause to many diseases and can be the reason for failure of pharmacotherapy (Padan and Landau, 2016; Clausen et al., 2017; Picci et al., 2022). Given the pivotal role of these transporters in cell homeostasis and health, a thorough understanding of their organization, functioning, and dynamics is highly desirable. A powerful tool to visualize the activity of ion transporters in living cells and reconstituted membrane systems are fluorescent compounds based on synthetic molecules. Although a variety of ion-targeting probes are commercially available, many have limitations in studies with membrane transporters. For example, water-soluble dyes such as pyranine (8-hydroxypyrene-1,3,6-trisulphonic acid) and fluorescamine (4-phenylspiro-[furan-2(3*H*),1-phthalan]-3,3′-dion) have been used to monitor the intravesicular pH values in studies of H^+^-translocating proteins (S. Li et al., 2011; Ohlsson et al., 2012; M. Li et al., 2015; Berg et al., 2017). However, their modest brightness (pyranine) and high bleaching rates (fluorescamine) make them difficult to use for pH measurements in vivo and in vitro. Rhodamine-based dyes provide an alternative due to their improved brightness and photostability; however, for reconstituted systems, problems with insufficient encapsulation during protein reconstitution and leakage out of vesicles were reported (Schubert, 2003; Leiding et al., 2009). More recently, membrane-bound pH sensors based on lipid-conjugated fluorophores have been utilized (Kemmer et al., 2015; Veshaguri et al., 2016; Schwamborn et al. 2017; Gerdes et al. 2018; Kühnel et al., 2019). These lipid-conjugated pH sensors efficiently co-reconstitute with membrane proteins into large liposomes, thereby avoiding loss during reconstitution and allowing studies in reconstituted systems down to the single vesicle level (Kemmer et al., 2015; Veshaguri et al., 2016; Kühnel et al., 2019). Lipid-conjugated pH sensors were also instrumental to study the activity of lipases on native substrate systems (Bohr et al., 2020). Furthermore, short-chain lipid-conjugated pH sensors enable monitoring of pH changes from neutral to acidic conditions in the endocytic pathway of living cells (Kühnel et al., 2019).

Here, we describe the synthesis of lipid-conjugated pH sensors from amine-reactive pHrodo esters and phosphatidylethanolamines. The method described here enables fast preparation of the sensor and can be used as a template to couple other amine-reactive fluorophores to phosphatidylethanolamines.

**Things to consider before starting**

- Choice of the fluorophore
  The fluorophore must contain an amine-reactive functional group such as NHS- or STP-ester, to allow for coupling with the ethanolamine head group of the lipid. Furthermore, the fluorophore characteristics must be suitable for the later use of labelled lipids, e.g., pH range to induce a change in fluorescence emission. In Table 1, a number of reactive fluorophores are listed that we coupled effectively to phosphatidylethanolamines by using the protocol described here.
- Choice of lipid
  The lipid headgroup must contain an ethanolamine group to allow for the coupling reaction with amine-reactive dyes. The lipid chains can vary. Herein, dioleoyl- and dipalmitoylphosphatidylethanolamine (DOPE and DPPE, respectively) were employed. The usage of other lipids might lead to decreased yields, as the reaction conditions are optimized for DOPE and DPPE.
- Choice of analysis method
  A method to verify the successful formation of the labelled lipid is e.g., high resolution mass spectroscopy (MS), since small amounts of substance can be reliably detected. Two potential methods are MALDI-TOF MS (matrix-assisted laser desorption/ionization with time-of-flight analysis MS) and ESI MS (electrospray ionization MS).
  **Table 1.** Fluorophores that have been coupled effectively to phosphatidylethanolamine (PE)

Fluorophore	Excitation (nm)	Emission (nm)	pH range#	p*K*_a_#
pHrodo Red NHS-ester	566	588	2.7–7.0	4.6
pHrodo Green STP-ester	505	543	2.7–7.0	4.6
SNARF-1 NHS ester	488–530	585/650†	6–11	9.3
Alexa Fluor^TM^ 488 NHS-ester	494	517	insensitive	---
TAMRA NHS-ester	541	567	insensitive	---
  **#**Values upon coupling to DOPE (Kemmer et al., 2015; Kühnel et al., 2019).
  †Values upon coupling to DOPE. Note that the emission spectrum of SNARF-1 undergoes a pH-dependent wavelength shift, thus allowing the ratio of the fluorescence intensities from the dye at two emission wavelengths to be used for more accurate determinations of pH.
  All catalog numbers provided below shall serve as guide; alternative sources can be used as well.

## Materials and reagents

1. Aluminium foil
2. Chromatography column, 200 × 10 mm, 15 mL (VWR, catalog number: 552-5410)
3. Disposable glass Pasteur pipettes (150 mm; VWR, catalog number: 612-1701)
4. Glass beads, 3 mm (Supelco, catalog number: 1040150500)
5. Glass cutter (Bohle, catalog number: BO 400.1)
6. Glass marbles (Fisher Scientific, catalog number: S04581)
7. Glass pipettes (e.g., graduated pipettes BLAUBRAND^®^ Type 3 Class AS, 10 mL, graduation: 10 mL; Carl Roth, catalog number: HXT8.1)
8. Glass wool (VWR, catalog number: 519-3101)
9. Hamilton 700 Series Syringes of 25, 100, and 500 μL (Peter Oehmen GmbH, catalog numbers: 9221013, 9221015, 6055335)
10. Long necked glass tubes (Carl Roth, catalog number: 0486.2)
11. Magnetic bars ROTILABO^®^ Micro, diameter 2 mm, length 5 mm (Carl Roth, catalog number: 0955.2)
12. Pipette tips 200 μL and 1,000 μL (SARSTEDT AG & Co. KG, catalog numbers: 70.760.002 and 70.3050.020)
13. Pointed-bottom glass tube, 10 mL (Merck, catalog number: CLS9950210)
14. Round-bottom glass tube, 10 mL, with joint and plug (Carl Roth, catalog number: NY90.1)
15. Spray bottle for primuline staining (Carl Roth, catalog number: YC44.1)
16. TLC plates, silica gel 60 (Merck, catalog number: 105626)
17. TLC chamber (Carl Roth, catalog number: 1N64.1)

**Chemicals**

1. Acetone p.a. (VWR, catalog number: 32201)
2. Ammonium molybdate tetrahydrate (Carl Roth, catalog number: 3666.1)
3. L-ascorbic acid (Carl Roth, catalog number: 3525.2)
4. Chloroform, ethanol-stabilized and certified for absence of phosgene and HCl (CHCl_3_) (Roth, catalog number: 7331.2)
5. CM Sepharose^TM^ Fast Flowing (Sigma-Aldrich, catalog number: 17 0719-01)
6. Deionized water
7. Dichloromethane (DCM) (VWR, catalog number: 32222)
8. N’,N’-Diisopropylethylamine (DIPEA) (Sigma-Aldrich, catalog number: D125806)
9. N’,N’-Dimethylformamide (DMF) (Sigma-Aldrich, catalog number: D4551)
10. Ethanol p.a. (VWR, catalog number: 20821.321)
11. Methanol (MeOH) (VWR, catalog number: 20847.307)
12. Nitrogen gas, 99.999% (ALPHAGAZ, Air Liquide, Düsseldorf, Germany)
13. Perchloric acid (VWR, catalog number: 20589)
14. Primuline (Sigma-Aldrich, catalog number: 206865)
15. Di-sodium hydrogen phosphate dihydrate (VWR, catalog number: 28029)
16. Triethylamine (VWR, catalog number: 8.08352)
17. Ascorbic acid solution (see Recipes)
18. Extraction solution (see Recipes)
19. Molybdate solution (see Recipes)
20. Phosphate standard solution (see Recipes)
21. Primuline staining solution (see Recipes)
22. Reaction solvent (see Recipes)
23. TLC eluent (see Recipes)

**Lipids**

1. 1,2-dioleoyl-*sn*-glycero-3-phosphoethanolamine (DOPE, Avanti Polar Lipids, Alabaster, AL, USA, catalog number: 850725)
2. 1,2-dipalmitoyl-*sn*-glycero-3-phosphoethanolamine (DPPE, Avanti Polar Lipids, Alabaster, AL, USA, catalog number: 850705)
*Note: This procedure has also been successfully performed using short-chain lipids including 1,2-dihexanoyl-sn-glycero-phosphoethanolamine (diC6-PE, catalog number: 850697), 1,2-dioctanoyl-sn-glycero-3-phosphoethanolamine (diC8-PE, catalog number: 850699), and 1-hexadecanoyl-2-hexanoyl-sn-glycero-3-phosphoethanolamine (C16C6-PE, custom made), all purchased from Avanti Polar Lipids (Alabaster, AL, USA).*

**Fluorescent dyes**

1. pHrodo Red NHS-ester (Invitrogen, catalog number: P36600)
2. pHrodo Green STP-ester (Invitrogen, catalog number: P35369)
3. Alexa Fluor^TM^ 488 NHS-ester (Invitrogen, catalog number: A20000)
*Note: This procedure has also been successfully performed using TAMRA NHS-ester (Lumiprobe, catalog number: 18120) and SNARF-1 NHS ester (Invitrogen, catalog number: S22801).*

## Equipment

1. Pipettes P200 and P1000 (GILSON^®^, catalog numbers: FD10005 and FD10006)
2. Analytical balance (Sartorius Entris-i II, 220 g/0.1 mg; Buch Holm, catalog number: 4669128)
3. Chemidoc MP imaging system (Bio-Rad) with illumination at 460–490 nm and 520–545 nm with 530/28 nm and 695/55 nm emission filters, respectively
4. CLARIOstar plate reader (BMG LABTECH)
5. Freezer -20 °C
6. Glass desiccator Boro 3.3 with socket in lid, 20 cm, including stopcock (BRAND GmbH, catalog number: 65238)
7. Heating block (Rotilabo^®^ Block Heater H 250; Carl Roth, catalog number: Y264.1)
8. Magnetic stirrer (e.g., IKAMAG^®^, DREHZAHL ELECTRONIC, IKA, Staufen im Breisgau, Germany)
9. Refrigerated centrifuge (e.g., Multifuge^®^ 3-R, Kendro Laboratory Products, catalog number: 75004371)
10. Vacuum Pump V-100 with interface I-100 and rotary evaporator Rotavapor^®^ R-100, SJ29/32, V, 220–240V (Buchi, catalog numbers: 11593636, 11593655D, and 11100V111, 11061895)
11. Vortex mixer (Vortex Genie 2 Scientific Industries Inc., catalog number: SI-0236)
12. Water bath (Julabo CORIO C-BT5, catalog number: 9011305)

## Software

1. ImageLab software version 5.2.1 (Bio-Rad)

## Procedure

- **Coupling reaction of DOPE with pHrodo Red NHS-ester (Figure 1)**
  Glassware should be used throughout the procedure, since lipids stick to plasticware, and chloroform can extract components from plasticware. Lipid stocks are handled on ice to reduce evaporation of chloroform during pipetting. In this section, the coupling of DOPE with pHrodo Red NHS-ester will be described. The procedure was also successfully performed using pHrodo Green STP-ester and Alexa Fluor 488 NHS-ester with DPPE as lipid. The labelling of short-chain lipids such as C_16_C_6_PE is also possible using this procedure.
*Note: The volumes given below serve just as an orientation. All volumes need to be calculated properly for each reaction depending on the used fluorophore and lipid.*
  **Figure 1.**
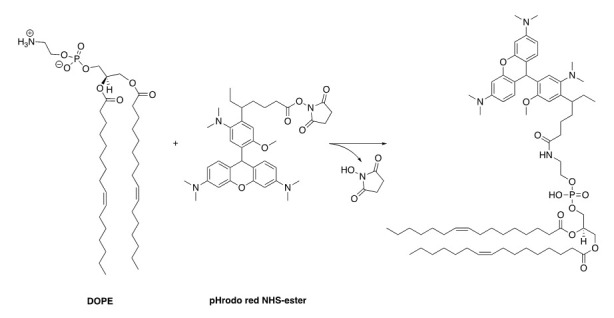
Synthesis scheme of pHrodo red–labelled DOPE
  1. Lipids are received in chloroform and packaged in sealed glass ampoules; store at -20 °C until use.
*Note: For long-term storage, evaporate the solvent in a vacuum desiccator and store the lipids at -80 °C to avoid oxidation of unsaturated lipids. To evaporate the solvent, we use a glass desiccator connected to a chemically resistant vacuum pump, reaching a final vacuum of 10 mbar (± 2 mbar) and a suction capacity of 1.5 m^3^/h.*
  2. To prepare lipid stocks, transfer 10 mg aliquots from the Avanti glass ampoule into glass screw neck vials. Evaporate the solvent from the glass vials in a desiccator at 250 mbar for 3 h with an additional incubation for 1 h at 30 mbar. Close the vials with screw caps and store at -20 °C until further use.
*Note: Chloroform is a hazardous solvent. Conduct all work in a fume hood while wearing proper protective clothing.*
  3. Remove desired lipid stocks from freezer, place on ice, and dissolve in chloroform to a final lipid concentration of 10 mg/mL.
*Note: Lipids other than DOPE may have limited solubility in chloroform and require a mixture of chloroform/methanol/water. Further directions and guidance can be found on the Avanti Polar Lipids’ web site (https://avantilipids.com/products).*
  4. Transfer the desired quantity of lipid (usually between 1 and 2 mg for 500 μg of fluorophore NHS-ester) from the CHCl_3_ stock solution to a round-bottom glass tube with a joint (NS 14). To maximize the reaction yield, we use a 2-fold molar excess of the lipid over fluorophore NHS- or STP-ester.
  5. Evaporate the solvent carefully under a N_2_ stream at room temperature. After solvent removal, add a small magnetic stirring bar.
*Note: We slide the large end of a disposable glass Pasteur pipette into laboratory tubing attached to the N_2_ gas cylinder. The flow is adjusted for a gentle stream of N_2_ gas through the pipette. The pipette can be held with a ring stand clamp to hold it in a fixed position.*
  6. Solubilize the fluorophore (500 μg) in 100 μL of the reaction solvent (see Recipes) and transfer it to the lipid-containing glass tube. Use approximately 100 μL for 500 μg of fluorophore.
  7. Add DIPEA to a final concentration of 130 mM (e.g., 2.3 μL of DIPEA for 100 μL of reaction solvent).
*Note: DIPEA is toxic and can cause severe skin burns.*
  8. Seal the vessel with a glass plug (NS 14) and wrap it in aluminium foil. Place the vessel on top of a magnetic stirrer and let the reaction stir overnight at room temperature.
- **Extraction of the labelled lipid**
  1. For extraction, add 0.5 mL of CHCl_3_, 1 mL of MeOH, and 0.5 mL of H_2_O followed by another 0.5 mL of CHCl_3_ and 0.5 mL of H_2_O. The reaction mixture should be monophasic at first and then turn biphasic (Figure 2).
    **Figure 2.**
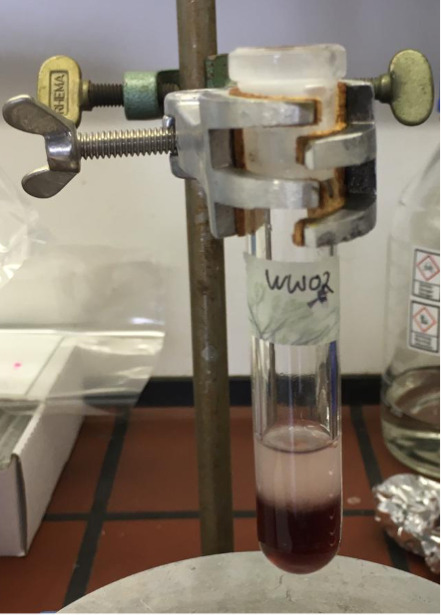
Result of the extraction process of fluorescent-labelled lipids. Coloured organic phase at the bottom and aqueous phase on top.
  2. Transfer the organic phase (coloured bottom phase) to a new glass tube.
*Note: Short-chain lipids may require several subsequent washes with CHCl_3_ to yield a decolorized aqueous phase.*
  3. Dry down the organic phase either by N_2_ stream or using rotary evaporator, depending on the volume of solvent.
  4. After complete evaporation of the solvent, either continue with purification (see Section C1 and C2) or store the sample at -20 °C.
- **Purification of the labelled lipid**
**C1. Purification via preparative thin layer chromatography (TLC)**
  1. Before starting the preparative TLC, the retention factor (R_f_) of the product needs to be determined. For this, a test TLC can be performed (Figure 3).
    - Prepare an approximately 5 cm wide TLC plate using a glass cutter.
    - Carefully draw a line approximately 1 cm above the bottom of the plate with a pencil.
    - Apply small volumes of the dissolved lipid stock and dissolved crude reaction product (e.g., with a glass Pasteur pipette) to the thin line at the bottom and let them dry completely.
    - Fill a TLC chamber with TLC eluent (see Recipes) up to a filling level of approximately 0.8 cm.
    - Close the lid and let the chamber saturate for approximately 5 min.
    - Place the TLC plate carefully and upright into the chamber and close the lid. Remove the TLC plate from the chamber when the solvent front is approximately 1 cm from the top.
    - Mark the solvent front line with a pencil.
    - Let the TLC plate dry under the fume hood for approximately 1 h before taking an image under white light and long-wave UV, to make fluorophore-coupled lipids and unreacted fluorophore visible using e.g., the Chemidoc MP imaging system.
    - Spray the plate with a primuline staining solution (see Recipes) to make lipids visible under long-wave UV light after drying.
    - Calculate the R_f_ value as follows:
$$R_{f}=\;\frac{Distance\;of\;product}{Distance\;of\;mobile\;phase}$$
      R_f_ values of different fluorophore-conjugated DOPE lipids are 0.70 and 0.55 for pHrodo Red-DOPE and pHrodo Green-DOPE, respectively.
    **Figure 3.**
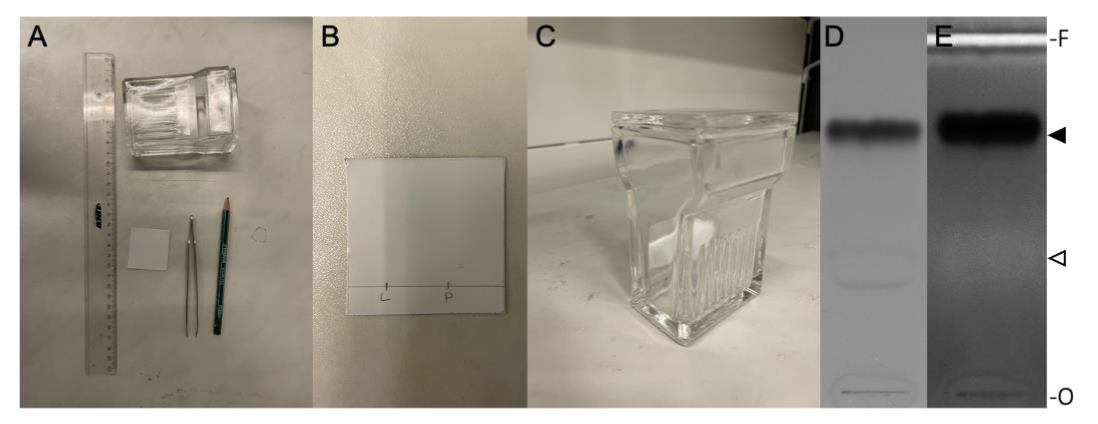
Thin layer chromatography (TLC). A. Materials needed for TLC preparation. B. Prepared TLC plate; L = pure lipid, P = reaction product. C. TLC plate placed upright in TLC chamber. D. TLC plate photographed under white light. E. TLC plate after primuline staining, photographed under long UV light. The position of non-reacted DOPE and pHrodo Red-DOPE is indicated by an open and filled arrowhead, respectively. O, origin; F, solvent front of the chromatograms.
  2. Prepare the preparative TLC by using a 20 cm long TLC plate.
    - Draw a line 1 cm above the bottom of a 20 cm long TLC plate.
    - Dissolve the reaction product in 100 μL of CHCl_3_:MeOH (2:1, v/v).
    - Apply the complete volume of dissolved product evenly on the line of the TLC plate, e.g., with a glass Pasteur pipette.
    - Let the TLC plate dry completely for approximately 1 h in the fume hood.
  3. Fill a TLC chamber with TLC eluent (see Recipes) up to a filling level of approximately 0.8 cm.
  4. Close the lid and let the chamber saturate for 20 min.
  5. Place the TLC plate upright in the chamber.
  6. Remove the TLC plate when the solvent front is approximately 1 cm beneath the top.
  7. Let TLC plate dry under the fume hood before taking an image under white light and under long-wave UV to make fluorophore-coupled lipids and unreacted fluorophore visible. A typical result is shown in Figure 4.
    **Figure 4.**
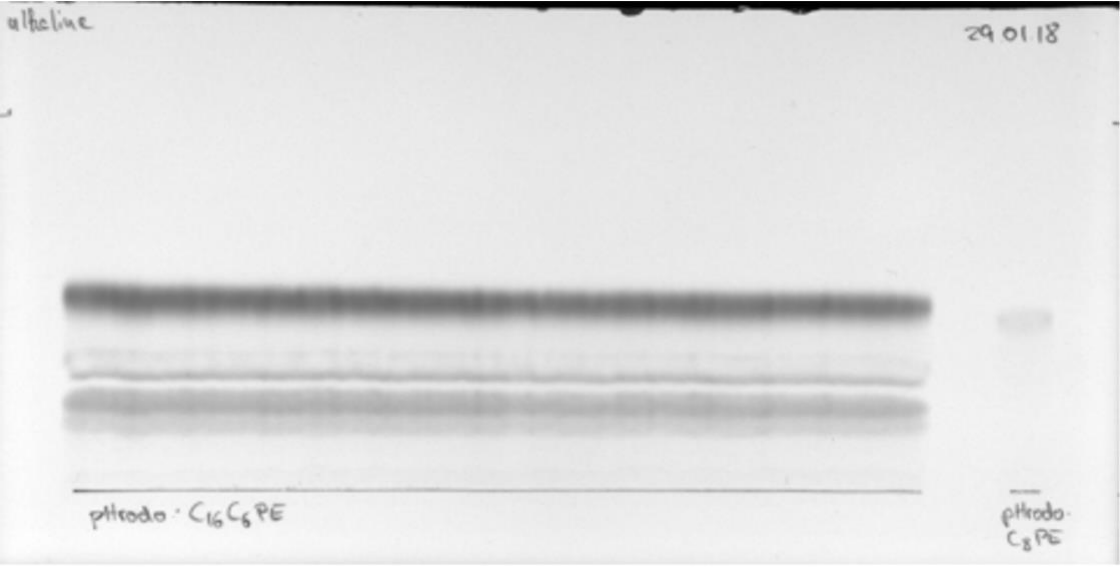
Preparative thin layer chromatography (TLC) of a pHrodo-labelled lipid with a reference of the pure product on the right side of the TLC plate. The chromatogram shown was dried completely before photographing under white light. The origin is indicated by the black line.
**C2. Re-extraction of the labelled lipid from preparative TLC**
  1. Carefully scrape off the product bands with a spatula and place the silica in pointed-bottom glass tubes (without joint, must fit into centrifuges).
  2. Add 1 mL of the extraction solution (see Recipes) to each tube used and vortex each tube for 1 min.
  3. Centrifuge the tubes at 480× *g* for 10 min at 8 °C.
  4. Transfer the liquid phase to a new glass tube.
  5. Repeat steps 2–4 until the liquid phase becomes colourless.
*Note: In the glass tube with the combined liquid phases, some silica might settle down at the bottom after a few minutes. The liquid phases can be transferred to a new vessel to remove silica.*
  6. To induce a phase separation, add a 1:1 mixture of CHCl_3_ and H_2_O (v/v). Transfer the organic phase (coloured bottom phase) to a new glass tube and dry down via N_2_ stream or in a desiccator. Proceed with quantification (Section D) or store at -20 °C.
**C3. Purification via column chromatography**
  As an example, the purification of Alexa Fluor 488 DPPE is used. The exact gradient conditions can vary depending on the used lipid and fluorophore.
  1. Prepare the column.
    - Provide the column with a glass wool plug to create a tight filter in front of the valve.
    - Transfer enough CM Sepharose (typically approximately 20–25 mL of suspension) into a beaker to yield a stationary phase of 10 cm height in the column (1cm diameter).
    - Fill the Sepharose into the column in one go and run out surplus MeOH.
*Note: Be careful to never let the column run dry!*
    - Close the valve before the top of the column runs dry. To remove air bubbles in the column, tap it gently e.g., with a cork ring while the Sepharose is settling.
    - After settling, cover the Sepharose under a 1–3 mm thick layer of glass beads. Gradually rinse the MeOH to CHCl_3_ according to the mixtures given in Table 2.
      **Table 2.** MeOH and CHCl_3_ ratios for rinsing the column

V_MeOH_ (mL)	V_CHCl3_ (mL)
7	3
5	5
3	7
1	9
0	10 (10×)
  2. Dissolve the reaction product in approximately 250 μL of CHCl_3_ and carefully add it to the top of the column by moving in a circle along the wall to ensure an even distribution. Wash your sample tube with small volumes until colourless and apply it on the column (Figure 5A).
  3. Perform the column chromatography while gradually increasing the MeOH content of the eluent, starting from pure CHCl_3 _(Table 3). Collect all fractions separately in 10 mL glass tubes.
  4. Evaporate the solvent in all sample tubes either in a desiccator or with a rotary evaporator. The reaction product can be identified via TLC (Figure 5B). Continue with quantification or store the samples at -20 °C.
    **Table 3.** Used MeOH and CHCl_3_ ratios for the purification of A488-DPPE

V MeOH (mL)	V CHCl_3 _(mL)	MeOH (%)	Total volume (mL)
--	20	0	20
1	19	5	20
1.4	18.6	7	20
4.5	45.5	9	50
3.6	26.4	12	30
9	51	15	60 (A488-DPPE)
15	15	50	30 (A488-DPPE)
20	10	67	30 (A488-DPPE)
    **Figure 5.**
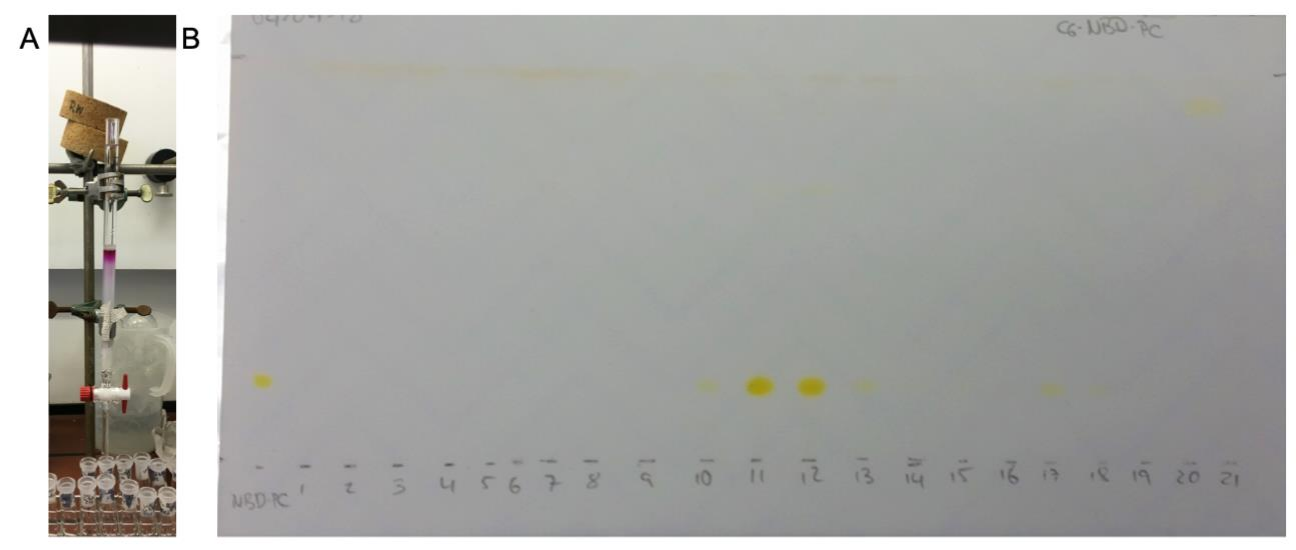
Purification via column chromatography. **A.** Used column for the purification of labelled lipids. **B.** TLC plate to determine the product containing fractions, exemplified for nitrobenzoxadiazole (NBD)-labelled lipid. The chromatogram shown was dried completely before photographing under ambient light.
- **Quantification of the reaction according to Bartlett (1959) and Ploier and Menon (2016), with small modifications to the protocol**
*Note: All steps described below must be performed in a fume hood using appropriate personal protection and following lab safety guidelines.*
  1. Turn on the electrical heating block (placed in fume hood) and set it to 195 °C.
  2. Prepare the sample tubes. We recommend preparing triplicates.
    - Dissolve the sample in an appropriate volume of CHCl_3_ and transfer approximately 20 nmol (assuming 100% yield) into a glass tube.
    - Evaporate the solvent via N_2_ stream.
  3. Prepare the standard tubes. We recommend preparing two standards per concentration.
    - Dilute an aliquot of the phosphate standard solution (see Recipes) to 0.4 mM in deionized water.
    - Transfer 0 (0 μL), 5 (12.5 μL), 10 (25 μL), 20 (50 μL), 30 (75 μL), 40 (100 μL), and 50 nmol (125 μL) phosphate to glass tubes.
  4. Add 650 μL of perchloric acid to all tubes (including standards) and cover them loosely with glass marbles.
  5. Heat all tubes in the heating block in the hood at 195 °C for approximately 1–2 h until the solution turns clear. Let the solution cool to room temperature.
  6. Turn on the water bath under the fume hood and set it to 80 °C.
  7. When samples are at room temperature, add 3.3 mL of deionized water to each test tube and vortex it.
  8. Add 0.5 mL of molybdate solution (see Recipes) to each test tube and vortex it.
  9. Add 0.5 mL of ascorbic acid solution (see Recipes) to each test tube and vortex it.
  10. Boil all samples at 80 °C in the water bath for 10 min.
  11. After cooling all the samples to room temperature, transfer each sample in duplicates to a 96-well plate.
  12. Determine the absorption of each well using a microplate reader set to 812 nm. The reading can be repeated at 780 and/or 720 nm to adjust for the best sensitivity.
  13. Average the duplicate and triplicate readings of each standard and sample, respectively.
  14. Subtract the value of the blank standard (0 nmol phosphate) from all standard and sample readings. This is the corrected absorbance.
  15. Plot standard curves (mean absorbance of wavelengths vs. nmol phosphate) and perform linear regression using e.g., Microsoft Excel, resulting in an equation of the type *y = mx + b*, where y is the absorbance, x is the phosphate concentration, and m is the slope; the intercept of the y-axis b is 0 after subtracting the value of the blank standard.
  16. Use the regression line to solve for sample concentration, by comparing the sample absorbance to the standard curve obtained (Figure 6).
    **Figure 6.**
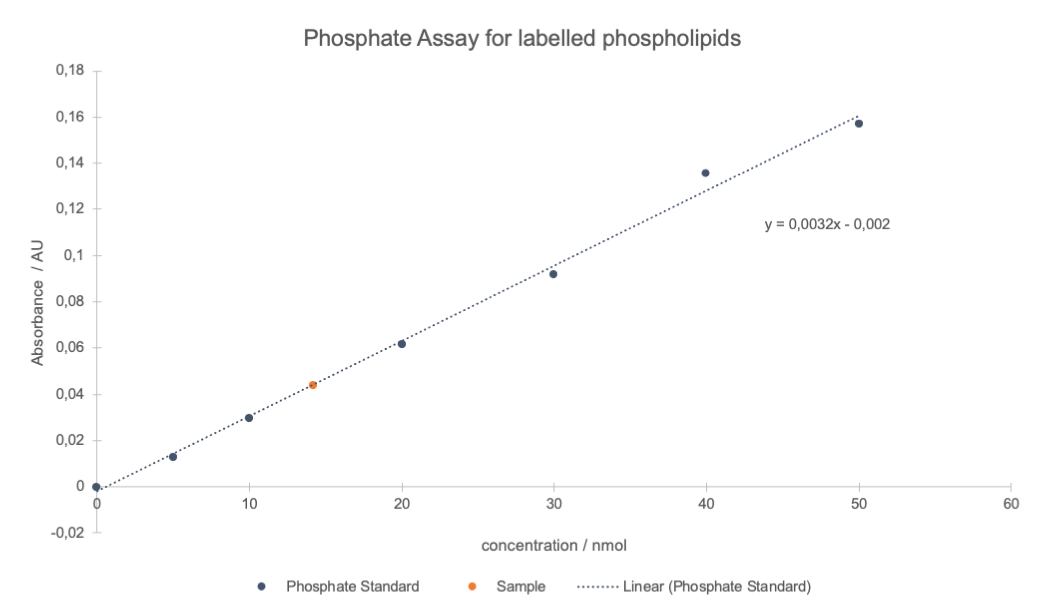
Typical plot of the results of a phosphate assay. Linear regression was applied to the averaged phosphate standard absorbance (812 nm) values of all different concentrations (blue points and dotted line). The sample concentration (red point) was determined using the equation given by linear regression and included into the graph.
  17. Analyse your product using reliable methods for small amounts such as mass spectroscopy. A typical result is shown for pHrodo Green-labelled DOPE in Figure 7.
    **Figure 7.**
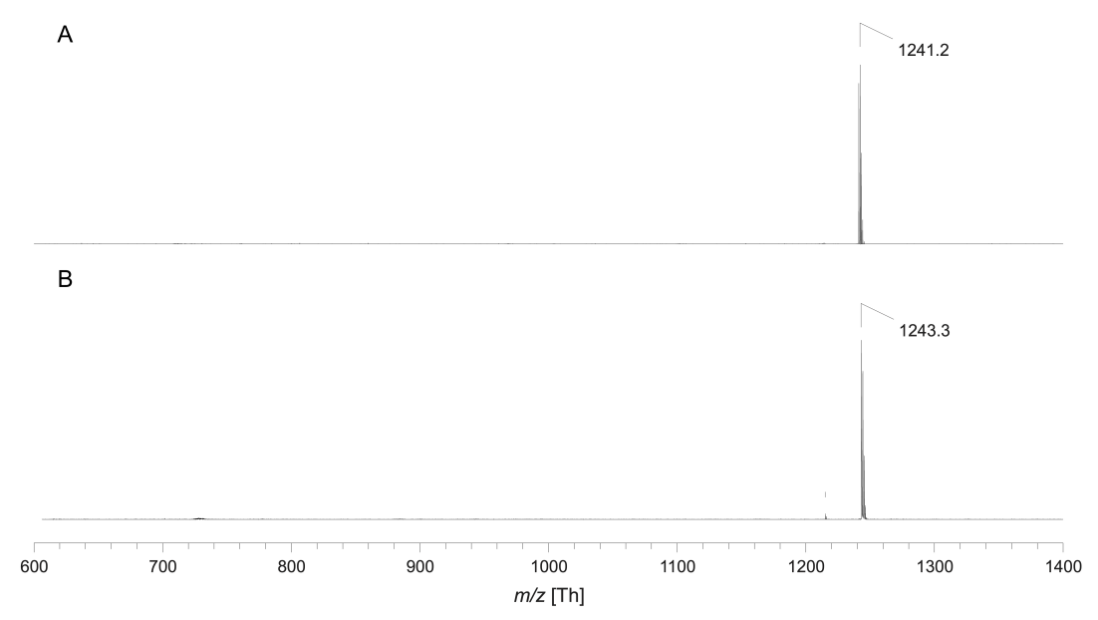
Exemplary MALDI-TOF mass spectra of pHrodo Green-labelled DOPE via MALDI-TOF mass spectrometry in negative ion mode (A) and positive ion mode (B). Since no chemical structure was published by the manufacturer and only an approximate molecular weight of ~750 g/mol was given, indicated signals correspond to the expected product peak in the range of mass per charge (m/z) 1,250. Accordingly, m/z 1,241.2 corresponds to the deprotonated product molecule and m/z 1,243.3 to the proton adduct.

## Recipes

1. **Ascorbic acid solution (50 mg m/L)**
  Dissolve 600 mg of L-ascorbic acid in 12 mL of deionized water. This solution cannot be stored.
2. **Extraction solution**
  Mix 2 mL of chloroform, 4.4 mL of MeOH, and 2 mL of H_2_O and store in a sealed glass bottle at room temperature.
3. **Molybdate solution (12 mg/L)**
  Dissolve 144 mg of ammonium molybdate tetrahydrate in 12 mL of deionized water. This solution cannot be stored.
4. **Phosphate standard solution (4 mM)**
  Dissolve 35.598 mg of di-sodium hydrogen phosphate dihydrate in 50 mL of deionized water. This solution can be stored at -20 °C.
5. **Primuline staining solution (0.05%, w/v)**
  Dissolve 50 mg of primuline in 100 mL of a mixture of water/acetone (v/v, 20:80)
6. **Reaction solvent**
  Mix 6 mL of DCM with 1 mL of DMF and store in a sealed glass bottle at room temperature.
7. **TLC eluent**
  Mix 30 mL of chloroform, 35 ml EtOH, 35 mL of triethylamine, and 7 mL of deionized water directly in the TLC chamber. This solution cannot be stored or used more than once.

## References

[1] Bartlett G. R.(1959). Phosphorus assay in column chromatography. J Biol Chem 234(3): 466-468. 13641241

[2] Berg J., Block S., Höök F. and Brzezinski P.(2017). Single Proteoliposomes with *E. coli* Quinol Oxidase: Proton Pumping without Transmembrane Leaks. Isr J Chem 57(5): 437-445.

[3] Bohr S. S., Thorlaksen C., Kühnel R. M., Günther-Pomorski T. and Hatzakis N. S.(2020). Label-Free Fluorescence Quantification of Hydrolytic Enzyme Activity on Native Substrates Reveals How Lipase Function Depends on Membrane Curvature. Langmuir 36(23): 6473-6481. 32437165 10.1021/acs.langmuir.0c00787

[4] Clausen M. V., Hilbers F. and Poulsen H.(2017). The Structure and Function of the Na,K-ATPase Isoforms in Health and Disease. Front Physiol 8: 371. 28634454 10.3389/fphys.2017.00371 PMC5459889

[5] Gerdes B., Rixen R. M., Kramer K., Forbrig E., Hildebrandt P. and Steinem C.(2018). Quantification of Hv1-induces Proton Translocation by a Lipid-couples Oregon Green 488-based assay. Anal Bioanal Chem 410(25): 6497-6505. 30027319 10.1007/s00216-018-1248-7

[6] Kemmer G. C., Bogh S. A., Urban M., Palmgren M. G., Vosch T., Schiller J. and Günther Pomorski T.(2015). Lipid Conjugated Fluorescent pH Sensor for Monitoring pH Changes in Reconstituted Membrane Systems. Analyst 140(18): 6313-6320. 26280031 10.1039/c5an01180a

[7] Kühnel R. M., Grifell-Junyent M., Jørgensen I. L., Kemmer G. C., Schiller J., Palmgren M., Justesen B. H. and Günther Pomorski T.(2019). Short-chain lipid-conjugated pH sensors for imaging of transporter activities in reconstituted systems and living cells. Analyst 144(9):3030-3037. 30901016 10.1039/c8an02161a

[8] Leiding T., Górecki K., Kjellman T., Vinogradov S.A., Hägerhäll C. and Årsköld S. P.(2009). Precise Detection of pH Insode Large Unimolecular Vesicles Using Membrane-Impermeable Dendritic Porphyrin-Based Nanoprobes. Anal Biochem 388(2): 296-305. 19248752 10.1016/j.ab.2009.02.023 PMC2804896

[9] Li M., Jørgensen S. K., McMillan D. G. G., Krzemiński Ł, Daskalakis N. N., Partanen R. H., Tutkus M., Tuma R., Stamou D., Hatzakis N. S., .(2015). Single Enzyme Experiments Reveal a Long-Lifetime Proton Leak State in Heme-Copper Oxidase. J Am Chem Soc 137(51): 16055-16063. 26618221 10.1021/jacs.5b08798 PMC4697922

[10] Li S., Hu P. C. and Malmstadt N.(2011). Imaging Molecular Transport across Lipid Bilayers. Biophys J 101(3): 700-708. 21806938 10.1016/j.bpj.2011.06.044 PMC3145269

[11] Ohlsson G., Tabaei S. R., Beech J., Kvassman J., Johanson U., Kjellbom P., Tegenfeldt J. O. and Höök F.(2012). Solute Transport on the Sub 100 ms Scale across the Lipid Bilayer Membrane of Individual Proteoliposomes. Lab Chip 12(22): 4635-4643. 22895529 10.1039/c2lc40518k

[12] Padan E. and Landau M.(2016). Sodium-Proton(Na^+^/H^+^) Antiporters: Properties and Roles in Health and Disease. In: Sigel, A., Sigel, H. and Sigel, R. K. O.(Eds.). *The Alkali Metal Ions: Their Role for Life*(pp. 391-458). Springer. 10.1007/978-3-319-21756-7_12 26860308

[13] Picci G., Marchesan S. and Caltagirone C.(2022). Ion Channels and Transporters as Therapeutic Agents: From Biomolecules to Supramolecular Medicinal Chemistry. Biomedicines 10(4): 885. 35453638 10.3390/biomedicines10040885 PMC9032600

[14] Ploier B. and Menon A. K.(2016). A Fluorescence-based Assay of Phospholipid Scramblase Activity. J Vis Exp(115): 54635. 27684510 10.3791/54635 PMC5092049

[15] Schubert R.(2003). Liposome Preparation by Detergent Removal. Meth Enzymol 367: 46-70. 10.1016/S0076-6879(03)67005-9 14611058

[16] Schwamborn M., Schumacher J., Sibold J., Teiwes N. K. and Steinem C.(2017). Monitoring ATPase Induced pH Changes in Single Proteoliposomes with the Lipid-coupled Fluorophore Oregon Green 488. Analyst 142(14): 2670-2677. 28616949 10.1039/c7an00215g

[17] Veshaguri S., Christensen S. M., Kemmer G. C., Ghale G., Moller M. P., Lohr C., Christensen A. L., Justesen B. H., Jørgensen I. L., Schiller J., .(2016). Direct Observation of Proton Pumping by a Eukaryotic P-type ATPase. Science 351(6280): 1469-1473. 27013734 10.1126/science.aad6429 PMC5023152

